# Timing of operation for poor-grade aneurysmal subarachnoid hemorrhage: study protocol for a randomized controlled trial

**DOI:** 10.1186/1471-2377-13-108

**Published:** 2013-08-19

**Authors:** Qiao Zhang, Lu Ma, Yi Liu, Min He, Hong Sun, Xiang Wang, Yuan Fang, Xu-hui Hui, Chao You

**Affiliations:** 1 Department of Neurosurgery, West China Hospital, Sichuan University, No.37 Guoxue Alley, Chengdu, Sichuan Province 610041, People’s Republic of China

**Keywords:** Timing of surgery, Poor-grade, Subarachnoid hemorrhage, ICP, Prognosis

## Abstract

**Background:**

Subarachnoid hemorrhage is a common and dangerous disease with an unfavorable prognosis. Patients with poor-grade subarachnoid hemorrhage (Hunt & Hess Grades 4–5) are unconscious on admission. Because of the high mortality and disability rate associated with poor-grade subarachnoid hemorrhage, it is often treated conservatively. Timing of surgery for poor-grade aneurysmal subarachnoid hemorrhage is still controversial, therefore this study aims to identify the optimal time to operate on patients admitted in poor clinical condition.

**Methods/design:**

Ninety-nine patients meeting the inclusion criteria were randomly assigned into three treatment groups. The early surgery group received operation within 3 days after onset of subarachnoid hemorrhage (day of SAH = day 1); the intermediate surgery group received operation from days 4 to 7, and surgery was performed on the late surgery group after day 7. Follow-up was performed 1, 3, and 6 months after aneurysm clipping. Primary indicators of outcome included the Extended Glasgow Outcome Scale and the Modified Rankin Scale, while secondary indicators of outcome were assessed using the Barthel Index and mortality.

**Discussion:**

This is the first prospective, single-center, observer-blinded, randomized controlled trial to elucidate optimal timing for surgery in poor-grade subarachnoid hemorrhage patients. The results of this study will be used to direct decisions of surgical intervention in poor-grade subarachnoid hemorrhage, thus improving clinical outcomes for patients.

**Trial registration:**

Chinese Clinical Trial Registry: ChiCTR-TRC-12002917

## Background

Subarachnoid hemorrhage (SAH) is a common fetal cerebrovascular disease. Its annual incidence is 2–32 cases per 100,000 population, and contributes to 5% of stroke cases [1,2]. More than 85% of SAH are caused by aneurysms [3]. Poor grade aneurysmal subarachnoid hemorrhage (PGASAH) , is classified as Grade IV and V and accounts for approximately 20-40% of patients with SAH [4]. The prognosis of PGASAH is extraordinarily poor. More than 60% patients will become dependent or will die [5,6]. Without surgical intervention, the mortality rate of PGASAH can reach more than 90% [7].

Neurosurgical clipping and endovascular coiling are considered two main treatments for patients with intracranial aneurysm. Whether patients with aneurysms can benefit from surgery is still controversial, especially for those with PGASAH, which is the most critical subtype of SAH. In 2003, Maurice-Williams RS asserted the history of aneurysm surgery would end with experienced neurosurgeons is draining away as a result of death and retirement [8]. However, the truth of the matter was quite different.

Previous trials have been performed to compare the prognosis of neurosurgical clipping and endovascular coiling. The most significant study is the International Subarachnoid Aneurysm Trial (ISAT) conducted by Molyneux et al. [9]. Two thousand one hundred forty three patients with aSAH were randomly divided into neurosurgical clipping (n = 1070) or endovascular treatment. Outcomes at 1 year indicated that although the rebleeding rate was more common after endovascular coiling than neurosurgical clipping, endovascular coiling resulted in better clinical outcomes.

However, some flaws existed in the ISAT study. First, optimal timing of surgery may not have been considered, because a 14 hours lapse in the intervention timing of the two treatments. Second, the study excluded more cases that were suitable for neurosurgical clipping. Third, the study didn’t include middle cerebral aneurysms. Fourth, long-term follow-up outcomes were unknown. Fifth, The ISAT study ignored a selection bias, with major surgical intervention centers in the United States and Japan being excluded [10]. Furthermore, the ISAT Collaborative Group distinctly known that the number of patients younger than 40 years or older than 70 years was small, no consistent trend for age was observed. And a limited number of patients with PGASAH were enrolled in the ISAT study [9]. So, the conclusion that patients with aSAH who received endovascular coiling showed a better clinical outcome than those received neurosurgical clipping should be suspected.

The timing of surgical intervention for SAH has been debated worldwide. Pros and cons exist for patients with PGASAH who received different timing of surgery. PGASAH patients are more prone to encounter rebleeding than patients in good clinical condition [11-14]. Early surgery can secure the aneurysm neck to avoid rebleeding which would cause irreparable outcomes, and reduce disability rate and mortality [6]. If the patient receives early surgery, triple-H therapy can be performed as soon as possible to avoid vasospasm postoperatively [14,15]. However, in early surgery, serious cerebral edema may cause brain injury, postoperative hemorrhage, difficult exposure of the aneurysm intraoperatively and other operative complications [16]. The advantages of delayed surgery include the less brain swelling and less cerebrovascular instability during the surgery. Decline in operative mortality have been associated with the operation that is relatively easy. However, the aneurysm may rupture again if surgery is delayed, thus increasing mortality [17].

Surgery timing for patients with PGASAH have the most uncertainty. Previous authors [5,18-21] carried out many researches on the surgery timing of PGASAH, yet a surgery timing still remains unclear. However, till now, only one prospective randomized study has been detected. Ohman et al. randomized 216 patients with SAH in clinical Grades I to III (according to the Hunt & Hess classification) into three operation groups: acute surgery (0 to 3 days after SAH), intermediate surgery (4 to 7 days after SAH), or late surgery (after day 7). At 3 months post-SAH, clinical outcomes indicated that there was no difference in the prognosis of the three groups [18]. In 2002, Ross et al. reported a study enrolled 1168 patients with SAH, and the outcomes indicated that there were no significant difference at discharge or 6 months between the early group (day 1–3), intermediate group (day 4–10) and late group (day 11–21). However, this study was only concerned with the prognosis of surgical patients, ignored the relationship between clinical grades, rebleeding and prognosis [19], the outcomes were widely suspected. Hutchinson et al. [5] reported their study with comparable outcomes, and their study contained similar shortfalls in analysis (as mentioned above). In 2002, de Gans et al. performed a systematic review, where only 11 studies, 1 randomized clinical trial and 10 observational studies, met the inclusion criteria [20]. Meta-analysis showed that outcomes were better after early or intermediate surgery than after late surgery, for patients who were in good clinical condition at admission. For patients in poor clinical condition, results suggest only a trend toward a better outcome for early or intermediate surgery when compared with late surgery. In 2005, Nieuwkamp et al. reported an observational study, which included 411 patients. The study demonstrated that clinical prognosis of patients in poor clinical condition on admission who received early surgery was statistically better than late surgery [21]. It is easy to detect that there is no randomized controlled trial concern about PGASAH.

The controversy over the optimum timing of surgery for patients with PGASAH has continued for many years, and evidence for the optimal timing of surgery in this condition is still insufficient. No randomized controlled trial specifically concerning PGASAH has currently been performed. We designed a trial to evaluate the prognosis of patients with PGASAH receiving surgery at different times, with the aim to identify optimal timing for surgical intervention.

## Methods/design

### Study objective

To compare the efficacy of different timing for surgical intervention in PGASAH. We will also attempt to screen patients with PGASAH who would benefit from surgical intervention.

### Overview of study design

West China hospital receives the most patients with PGASAH in western of China. This study is a prospective, single-centre, observer-blinded, randomized controlled trial with stratification according to the patient’s age, gender, Hunt & Hess grading and the size of aneurysm. Ninety-nine patients meeting the criteria will be randomly assigned into three treatment groups. The early surgery group will receive operation within 3 days after onset of SAH, the intermediate surgery group from days 4 to 7, and the late surgery group after day 7 post-SAH.

### Sample size

The rate of unfavorable outcomes in patients with PGASAH is approximately 50% [22]. A statistically significant difference in unfavorable outcomes between the different surgery times is 30%, which would have statistically significant differences. A sample size of 88 will be required with 80% power and a 5% (2-sided) significance level. On consideration of the loss to follow-up, the sample size will be increased to 99 subjects.

### Inclusion criteria

● Age between 18 to 80 years old.

● Patient diagnosed with a definite subarachnoid hemorrhage, using computed tomography (CT) or lumbar puncture.

● Patient had at least one intracranial aneurysm, detected by DSA, MRA or CT angiography, which was responsible for the recent subarachnoid hemorrhage.

● Patient was in poor neurological condition (Hunt & Hess Grades IV or V) on admission.

● No more than 72 hours from onset to admission.

● According to ethics committee requirements, the person responsible for the patient must agree to participate in this trial and sign the informed consent.

### Exclusion criteria

● Pseudoaneurysm [23].

● Drug addiction.

● Patients with the following systemic diseases:

   serious coagulation dysfunction

   organ failure

   serious psychosomatic diseases

● HIV.

● Pregnant or lactating women.

● The person responsible for the patient refused to give consent to participate in this trial.

● Patient was participating in another randomized clinical trial for treatment of SAH.

### Withdrawal from the study

● Even if patients are included in the trial, the person responsible for the patient can withdraw informed consent at any time.

● If severe adverse events occur, patients can be withdrawn from the study on consideration of their personal safety.

● If the person responsible for the patient decides to abandon treatment, patient will be withdrawn as they do not fully participate in the trial.

### Randomization

Minimization, a kind of balance randomization method, will be used in this trial. Characteristics of patients who meet the selection criteria will be submitted to random analyst. After the data is entered into a computer, patients will be randomly divided into an early, intermediate or late surgery group using specialized minimization software. Once random allocation has been determined, groups cannot be changed. Stratified randomization will be employed. Four stratification factors will be applied to achieve balanced randomization: 1) patient’s age, 2) gender, 3) Hunt & Hess grading and 4) the size of aneurysm.

### Blinding

Because of the nature of the study, only the observer can be blinded. In order to prevent bias, the following measures will be taken. 1) Patient characteristics will be collected and evaluated by two special doctors who are not involved in any other stage of the trial. 2) Patient randomization will be performed by an independent random analyst. 3) Patient outcomes will be followed up by two special investigators who are not involved in the allocation or the treatment process. 4) Statistical analysis will be performed by a special statistician who did not participate in the implementation of the study. 5) Because of the sequence of the Case Report Form (CRF), each stage of the study will be completed in a specific order by the allocated individuals. Patient characteristics will be the first section completed, followed by the independent random analyst, subsequently the neurosurgeons will describe the treatment procedure, complications, adverse and severe adverse events in the CRF, the last will be the two special investigators. All the researchers will submit a copy of their original data to the Quality Monitoring Board (QMB), before the CRF is available.

### Treatment

All patients with SAH admitted in poor neurological condition (Hunt & Hess IV–V) will receive effective treatment, according to guidelines for the management of aSAH that published in 2012[24]. Aggressive resuscitation, including intubation, ventilation, and mannitol administration, will be implemented in the emergency department. CT scan will be performed as soon as possible after admission. Intracranial pressure (ICP)-monitoring sensor implantation (Codman Group Inc) will be performed on all identified patients after admission to continuously measure ICP. If aSAH-related hydrocephalus is confirmed by CT scan, both external ventricular drain (EVD) and ICP-monitoring sensor implantation will be performed. ICP-targeted treatment will be given to patients with elevated ICP. These therapeutic measures include head elevation to 30°, hyperosmolar therapy, tracheal intubation, sedation and analgesia, and mild hypothermia. Neurosurgical clipping will be performed at different times, according to the randomized grouping. Aneurysms of the anterior circulation were approached through the standard pterional craniotomy and all aneurysms were obliterated by direct clipping. Postoperatively, patients will return to the neurosurgical intensive care unit (NICU) for intensive monitoring.

### Follow-up

Two investigators (senior neurosurgeons) that are not involved in allocation or treatment will follow up of all patients at 1, 3, and 6 months after neurosurgical clipping. Primary outcomes of the trial are the Extended Glasgow Outcome Scale and the Modified Rankin Scale (mRS) at 6 months after aneurysm clipping. Both scales will be measured with structured interviews. The secondary indicators include the Barthel Index and mortality.

### Data collection and handling

All relevant information will be collected using a case report form (CRF). Double data input will be used for the data entry, and will be checked at the second input. The QMB and the principal investigator will check and examine the data, which will then be secured and sent to the statistician.

### Ethical issues

The final study protocol and the final of written informed consent form were approved by the Biological and Medical Ethics Committee (BMEC) of West China Hospital (2012 Reviewed-No.114). Good Clinical Practice and ethical principles described in the Declaration of Helsinki will be strictly adhered to guarantee patient rights. The trial will be carried out in keeping with local laws and regulations. Full disclosure will be given to the person responsible for the patient. The information will be given before the person responsible agrees to participate in this trial and signs the informed consent, and will include the natural history of SAH, the treatment procedure, risks, possible consequences and the costs of PGASAH. Patients can be withdrawn from the trial for any reason and at any time.

### Adverse and severe adverse events

Adverse events (AE) are defined as any unwanted incidents in patients during the trial. Coma was excluded as an AE, because all patients enrolled in the trial were in poor neurological condition on admission. Serious adverse events (SAE) are defined as death or vegetative state. Both AEs and SAEs will be recorded on the CRF, including the details of the event, the correlation with the trial, the start and the end date, treatments used, their efficacy and outcomes. In the case of AEs and SAEs, the BMEC and QMB will be informed within 24 hours after the SAEs are detected .

### Statistical analysis

Intention-to-treat (ITT) analysis and per-protocol (PP) analysis will be performed. The level of significance required for 80% power and a 5% (2-sided) significance level will be used. All data will be analyzed by the statistical analysis using Statistical Package for Social Sciences software (SPSS), version 17.0. Quantitative variables will be analyzed using t-test. Chi-square test and Fisher’s exact test will be used to analyze categorical variables. To analyze the outcome at 6 months after surgery, a comparison between groups will be made using the Mann–Whitney U-test and the significance of observed differences in outcome will be assessed with the Fisher’s exact test. Survival analysis will be further compare mortality between groups. Logistic regression will be used to adjust the effect of multivariable.

## Discussion

Many neurosurgeons are reluctant to treat patients with PGASAH, because the disease burden higher mortality, higher disability, more medical care costs, and higher medical risks. Although neurosurgery has rapidly developed in recent years, the prognosis of PGASAH remains dismal. Controversy surrounding the optimal timing of surgery for PGASAH still continues. In spite of many difficulties, we carried out the trial on the consideration of the following reasons: First, although many neurosurgeons have done much work on the timing of surgery for PGASAH, there is a lack of randomized controlled trial focusing specifically on this condition. Second, no optimal timing, no better surgical intervention outcome. The prognosis for PGASAH is poor, owing to the nature of the disease, but also contributed to the timing of surgery. Third, there has been great progress both in neurosurgical clipping and endovascular coiling. The comparability of treatment outcome between neurosurgical clipping and endovascular coiling should be suspected, if the outcome of PGASAH that yield by the optimal surgical timing is lacking. In a similar way,the lack of optimal timing for coiling [25] may prevent the clinical outcomes of these two treatments from being comparable.

**Figure 1:**
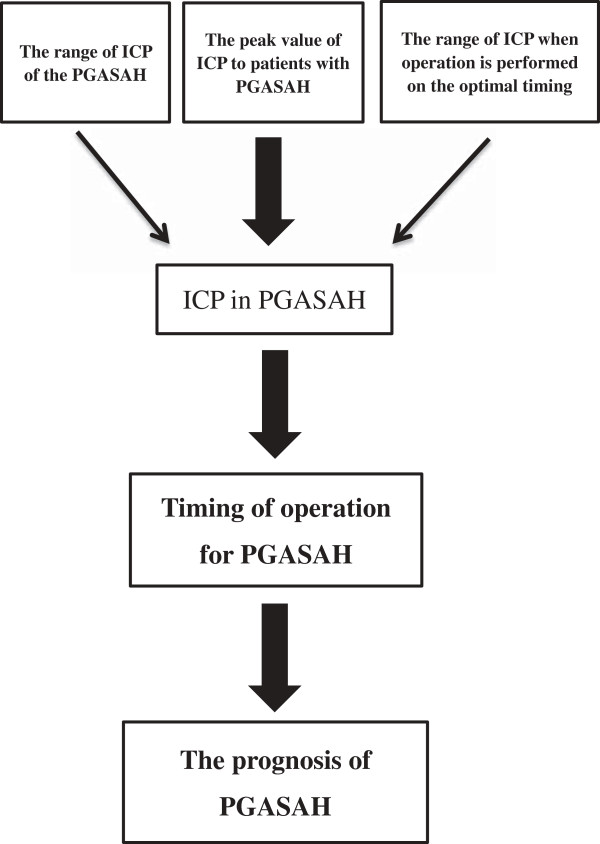
The study considerations of our trial.

As we know, the prognosis of PGASAH is affected by many factors. The timing of surgery for PGASAH plays an important role in patient outcomes. The factors that cause the initial poor condition of patients with PGASAH are still not clear. Few studies have focused on ICP of the PGASAH. What is the range of ICP in PGASAH? And whether there is a relationship between the elevated intracranial pressure (EICP) and the PGASAH? The peak value of ICP that patients with PGASAH can withstand, the duration of this EICP and the ideal ICP in optimal timing for surgery are all variables we wish to elucidate from our study. The study considerations of our trial are presented in Figure 1.

This trial was elaborately designed to avoid several forms of bias and improve accuracy. The experience of presciously authors was noted and was merged into our design of the trial [19-21]. We will record all treatment results and surgical results, but only treatment results will be utilized for analysis. This will avoid selection bias, because all treatment results will include patients that may die from rebleeding, vasospasm or other complications, when awaiting surgery, especially those patients assigned to the intermediate or late surgery groups. Patients in intermediate or late surgery groups will receive emergency surgery when space-occupying hematoma, midline shift and emergency surgery indications are confirmed. Bias will be solved through the use of ITT analysis.

Although previous reports have demonstrated that patients in poor condition on admission within 6 hours after the onset of SAH achieved better outcomes than patients admitted later [12,26], this trial will enroll all the patients with PGASAH on admitted within 72 hours. As one of the largest hospitals in China, the number of patients admitted within 6 hours after the onset of SAH was relatively small in our department. More patients are transferred from other cities, even from the outlying areas in the west of China, and which takes time. The trial was not designed to include a small number of patients, but to include the overwhelming majority of the patients.

Neurosurgeon experience is an important factor in outcome after SAH. More than 4,000 surgeries are completed in our Department of Neurosurgery every year. Every neurosurgeon participating in the trial has accumulates experience of more than 200 neurosurgical clippings yearly, thus ensuring that every surgery will be performd to a high standard.

According to previous report, the major risk of rebleeding after SAH presented within the first 6 to 12 hours, and the risk of ultra-early rebleeding was even higher for patients with PGASAH [10]. Many neurosurgeons attempted to performed early or ultra-early/urgent surgery on the patients with PGASAH [21,27], so that prevented rebleeding. However, some authors detected the incidence of rebleeding with a higher frequency [28,29], even as high as 89% [30], during emergency cerebral angiography within 6 hours after initial SAH. As we know, the frequence of rebleeding will increase during cerebral angiography, because the thrombus at the aneurysm dome is still fragile only a few hours after initial SAH, and the injection of contrast medium may suddenly increase the intra-arterial pressure. Indeed, ultra-early surgery did not improve the rebleeing rates and clinical outcomes of patients [31,32]. Therefore, whether the patients with PGASAH benefit from early or ultra-early/urgent surgery is still unclear.

In conclusion, evidence for the optimum timing of surgery is still insufficient. We will evaluate the prognosis of patients with PGASAH receiving surgery at different times, aiming to identify optimum timing for intervention in these cases.

## Abbreviations

SAH: Subarachnoid hemorrage; PGASAH: Poor grade aneurysmal subarachnoid hemorrhage; EVD: External ventricular drain; GOSE: Extended glasgow outcome scale; mRS: Modified rankin scale; BMEC: Biological and medical ethics committee; CRF: Case report form; QMB: Quality monitoring board; CT: Computed tomography; DSA: Digital subtraction angiography; MRA: MR angiography; CTA: CT angiography; AE: Adverse events; SAE: Serious adverse events; ITT: Intention to treat; SPSS: Statistical package for the social sciences; EICP: Elevated intracranial pressure.

## Competing interests

The authors declare that they have no competing interests.

## Authors’ contributions

CY is the principle investigator, originally conceived the idea for the study. QZ, LM, YL, MH, HS, XW, YF, XH and CY all participated in the design of the study and selection of outcome measures. QZ and LM have been part of the trial design and drafted the protocol. All authors have read, edited and approved the final manuscript.

## Pre-publication history

The pre-publication history for this paper can be accessed here:

http://www.biomedcentral.com/1471-2377/13/108/prepub
